# Suboptimal Iodine Status and Low Iodine Knowledge in Young Norwegian Women

**DOI:** 10.3390/nu10070941

**Published:** 2018-07-21

**Authors:** Sigrun Henjum, Anne Lise Brantsæter, Astrid Kurniasari, Lisbeth Dahl, Eli Kristin Aadland, Elin Lovise Folven Gjengedal, Susanne Birkeland, Inger Aakre

**Affiliations:** 1 Department of Nursing and Health Promotion, Faculty of Health Sciences, OsloMet—Oslo Metropolitan University, 0310 Oslo, Norway; astrid.kurniasari@medisin.uio.no; 2 Division of Infection Control and Environmental Health, Norwegian Institute of Public Health, 0403 Oslo , Norway; AnneLise.Brantsaeter@fhi.no; 3 Institute of Marine Research (IMR), P.O. Box 1870 Nordnes, 5817 Bergen, Norway; lisbeth.dahl@hi.no (L.D.); inger.aakre@hi.no (I.A.); 4 Faculty of Education, Western Norway University of Applied Sciences, 5063 Bergen, Norway, Eli.Kristin.Aadland@hvl.no; 5 Faculty of Environmental Sciences and Natural Resource Management, Norwegian University of Life Sciences, Aas 1433, Norway; elin.gjengedal@nmbu.no (E.L.F.G.); susanne.birkeland@nmbu.no (S.B.)

**Keywords:** iodine status, young women, pre-pregnancy, iodine deficiency, iodine intake, urinary iodine concentration, knowledge on iodine, Norway

## Abstract

Previous studies have documented mild to moderate iodine deficiency in pregnant and lactating women in Norway. This study focused on non-pregnant young women because their future children may be susceptible to the adverse effects of iodine deficiency. We assessed urinary iodine concentration (UIC), iodine intake from food and supplements, and iodine knowledge in 403 non-pregnant women, mainly students, aged 18–30 years. Iodine concentration was measured in spot urine samples analyzed by inductively coupled plasma mass spectrometry and iodine intake was calculated from a self-reported food frequency questionnaire. Knowledge about iodine was collected through the self-administered, paper-based questionnaire. Median (p25–p75) UIC was 75 (42–130) µg/L and 31% had UIC < 50 µg/L. Habitual iodine intake was 100 (69–136) µg/day. In multiple regression models, supplemental iodine, use of thyroid medication, and iodine intake from food were positively associated with UIC, while vegetarian practice was negatively associated, explaining 16% of the variance. Approximately 40% of the young women had low iodine knowledge score and no differences were found between women in different study programs. Young women in Norway are mild to moderately iodine-deficient, and public health strategies are needed to improve and secure adequate iodine status.

## 1. Introduction

Iodine deficiency has substantial effects on growth and development and is the most common cause of preventable mental impairment worldwide [1,2]. Maternal thyroxine is crucial for maturation of the fetal nervous system, especially for the development of the fetal thyroid before 18–20 weeks of pregnancy [3,4], and even mild to moderate iodine deficiency could be harmful [5,6,7,8,9]. As sufficient supply of maternal thyroid hormones are crucial in early gestation, iodine deficiency should be corrected pre-pregnancy [10]. Mild-to-moderate iodine deficiency occurs in areas that have not previously been recognized as iodine-deficient; 45% of the population of continental Europe shows evidence of iodine deficiency [11]. Norway has been considered iodine-replete for decades as a consequence of iodine fortification of cow’s fodder and high milk consumption. However, recent studies have documented suboptimal iodine status in pregnant women [8,12,13], lactating women [14], elderly [15], vegans [15], and non-pregnant women of childbearing age [15,16]. There are few dietary iodine sources in Norway; the only good sources are milk (contributing ~60%) and seafood (contribution ~15%) [17,18]. Other foods such as eggs and whey cheese contribute small amounts, while household salt and drinking water contribute negligible amounts [15,17]. The consumption of milk and dairy products is declining, while plant-based milk alternatives are becoming increasingly popular. Individuals with no or low intake of cow’s milk will not get sufficient iodine unless they use iodine-containing supplements. In a report on iodine status in Norway, iodine intake was estimated; iodine intake in men was within recommended levels and significantly higher than among women. The women in the youngest age group (18–25 years old) had the lowest iodine intake [16]. Previous studies suggest that lack of information and knowledge about iodine may be a major risk factor for iodine deficiency [19,20,21,22]. Nothing is known about iodine knowledge in young Norwegian women. Ensuring sufficient iodine intake in young women is particularly important as their iodine store is of crucial importance for fetal development once they proceed to pregnancy [1,23]. In Norway, there is limited knowledge about iodine status in young women and the National Nutrition Council has expressed a special concern for this vulnerable group [16]. The aim of this study was to evaluate iodine status and iodine knowledge by assessing urinary iodine concentration, iodine intake, and iodine knowledge in non-pregnant young women.

## 2. Materials and Methods

### 2.1. Population and Study Design

Convenience sampling was used to recruit 403 young non-pregnant women, aged 18–30 years, with no previous children, from two different geographical regions in Norway (eastern and western Norway). The invitation to participate in the study was given to university students attending lectures or classes. All young women interested in the project met with project workers and received information about the study purpose. Those willing to participate gave informed written consent. Young employees at the study sites who fulfilled the inclusion criteria were also allowed to participate (*n* = 8). The data collection took place from September to December 2017 in eastern Norway, and from January to February, 2018 in western Norway. To be eligible for the study, participants had to be able to read and write Norwegian. Participants were asked to answer a questionnaire covering information about age, anthropometrics, demographics, health, study program, smoking habits, iodine knowledge, vegetarian dietary practice, and habitual intake of food intake and dietary supplement use. In addition, they were asked to provide a spot urine sample. The present study was conducted according to the guidelines in the Declaration of Helsinki and was approved by the Regional Committee for Medical and Health Research Ethics Norway (2015/1845).

### 2.2. Urinary Iodine Concentration

Participants provided a non-fasting (random) spot urine sample in a labelled 100 mL Vacuette^®^ Urine beaker (Greiner Bio-One, Kremsmünster, Austria). The urine samples were stored at <4 °C prior to handling. At the laboratory, the spot urine samples were stored at –80 °C pending analysis. Aliquots of 1.00 mL urine were transferred into 15-mL polypropylene (pp) centrifuge tubes (Sarstedt, Nümbrecht, Germany) by means of a 100–5000 µL electronic pipette (Biohit, Helsinki, Finland) and subsequently diluted ten times with an alkaline solution (BENT), containing 4% (w/V) 1-Butanol, 0.1% (w/V) H_4_EDTA, 2% (w/V) NH_4_OH, and 0.1% (w/V) Triton X-100. Reagents of analytical grade or better and deionized water (>18 MΩ) were used throughout. The quantification of iodine was performed by means of the Agilent 8800 Triple Quadrupole ICPMS (Agilent, Santa Clara, CA, USA) using oxygen reaction mode. Iodine was determined on mass 127. ^129^I was used for correction of non-spectral interferences. Certified reference materials (CRM) used for quality control were Trace Elements Urine L-1 (78 µg/L) and Trace Elements Urine L-2 (280 µg/L) from Seronorm^TM^ (SERO AS, Billingstad, Norway), and SRM 2670a Toxic Elements in freeze-dried urine from The National Institute of Standards and Technology (NIST, Gaithersburg, MD, USA) (88.2 µg/L). All the measured values of CRM were within the certified ranges. The same procedure was followed for blank samples as other samples, and all had values under the limit of detection (LOD, 0.4 µg/L) or limit of quantification (LOQ, 1.2 µg/L). The detection and quantification limits were calculated at three and ten times the standard deviation (SD) of blank samples, respectively. Intermediate precision (within-laboratory reproducibility) was <4% [24]. The Norwegian University of Life Sciences in Aas (Faculty of Environmental Sciences and Natural Resource Management) performed measurement of the iodine concentration.

### 2.3. Assessment of Vegetarian Dietary Practice and Iodine from Food and Dietary Supplements

The questionnaire included specific questions about habitual and recent (last 24 h) use of dietary supplements and intake of milk and yoghurt. Furthermore, participants were asked to report their overall dietary profile according to four alternative answers specifying exclusion/inclusion of meat, milk, eggs, and fish (including products derived from these). The alternatives were: (1) ovo-lacto (OL) vegetarian, i.e., exclusion of meat and fish, but not eggs and milk; (2) ovo-lacto-pesco (OLP) vegetarian, i.e., exclusion of meat, but not fish, eggs and milk; (3) ovo-pesco (OP) vegetarians, i.e., exclusion of meat and milk, but not fish and eggs, and (4) vegans, i.e., exclusion of all animal products.

The last page of the questionnaire was designed as a food frequency questionnaire asking the participants to report their habitual food intake by answering 32 questions about average intake during the last four weeks of selected food items/dishes. Of these, four questions assessed intake of milk, yoghurt, and cheese, four assessed intake of fish and fish dishes, and one assessed intake of eggs and egg dishes. The questions had seven answer alternatives, ranging from rarely/never to five times daily or more. The answers to the questions related to intake of milk, cheese, fish, and eggs were converted to daily amounts and multiplied with the iodine concentration for each food item/dish using values in the Norwegian Food Composition Table. We used recipes to derive the iodine concentrations in composite dishes containing milk, fish, and eggs and for averaging concentrations from different fish species. To account for iodine contributed by the remaining food items which assessed aggregated food items with low iodine concentrations (e.g., vegetables, fruits, drinks), we added 30 µg/day to each estimated total intake. This approach was also used in previous studies [12,14].

For assessing the amount of iodine contributed by dietary supplements, participants were asked to report habitual as well as recent (last 24 h) use of all dietary supplements. Each supplement was to be reported by brand name and how many times weekly the supplement was taken. Using information provided by producers and labels, we calculated the daily amount of iodine contributed by iodine-containing supplements and added this to the calculated 24-h and iodine intake from food to obtain habitual iodine intake.

### 2.4. Assessment of Iodine Knowledge

The first part of the questionnaire included five questions on iodine knowledge. These questions were adopted from a questionnaire used in the UK [20] and adapted to Norwegian after a content validation by three subject experts and a pilot test among four pregnant women (for further information see [22]). The questions used in this study were: (1) Do you know what iodine is? (2) What are the most important dietary sources of iodine? (3) Why is iodine important? (4) What do you know about the current iodine status in Norway? (5) Do you feel confident that you achieve the daily requirement for iodine? The iodine knowledge questions (2, 3, and 4) were used to calculate an iodine knowledge score. These three questions had multiple answer alternatives, some correct and some incorrect. Correct answers generated 2 points, correctly identified false answers generated 1 point, and incorrect answers gave 0 points. The total knowledge score ranged from 0–24 points and was divided into four categories: poor knowledge (0–6 points), low knowledge (7–12 points), medium knowledge (13–18 points), and high knowledge (19–24 points). The scale had acceptable reliability and internal consistency with standardized Cronbach’s alpha of 0.59, which is considered acceptable for unidimensional scales [25]. Exploratory factor analysis identified one factor only, confirming unidimensionality. The factor loadings were 0.80 for ‘iodine sources’, 0.76 for ‘iodine importance’ and 0.67 ‘for ‘iodine status in Norway’, reflecting high correlation with the underlying factor.

We examined iodine knowledge scores according to the study program of the participants, i.e., health sciences vs. other sciences, and across both groups. The following study programs were included in health sciences group: nutrition, public health nutrition, nursing, public health (nursing), midwifery, social nursing, physiotherapy, occupational therapy, and mental health work. Other sciences included: biotechnology, chemistry, environmental sciences, engineering, physics, teaching, psychology, and other. Due to lack of a validated cut-off for the iodine knowledge score, the score was categorized at the 66th percentile for the statistical analyses, which was decided prior the analyses. We also examined whether iodine knowledge was associated with iodine intake.

### 2.5. Definitions of Iodine Status and Recommendations for Iodine Intake

In this study, we apply the epidemiological criteria’s for assessment of iodine nutrition established by World Health Organization (WHO) [1]. The recommended indicator for evaluation of iodine status is the population median UIC, and median UIC below 100 µg/L is considered to reflect insufficient iodine intake in non-pregnant women. The Nordic Nutrition Recommendations stipulate a daily iodine intake of 150 µg/day for non-pregnant women of reproductive age, and the average requirement is 100 µg/day [26].

### 2.6. Statistical Methods

Data were analyzed using IBM SPSS statistics version 23 (IBM Corp., Armonk, NY, USA) and STATA 14 (StataCorp, College Station, TX, USA). Normally distributed data were presented as mean ±SD and non-normally distributed data as median and 25th–75th percentile (p25–p75). We used Mann Whitney U test or Kruskall Wallis to examine differences in continuous variables between groups and Spearman correlation to examine agreement between continuous variables. UIC was not normally distributed. Predictors of UIC were identified using median regression (quantile regression). Median regression is less sensitive to outliers than mean regression and non-transformed UIC can be used as the dependent variable. The beta coefficients represent the estimated change in the median UIC, conditional on the values of the independent variables [27]. Background characteristics and iodine intake associated with UIC in bivariate analyses (*p* < 0.2) were examined as independent variables. These were iodine intake from food, iodine supplement use, thyroid disorder, vegetarian diet, and smoking habits. Iodine knowledge score categorized at the 66th percentile (0 ≤ 66th percentile, 1 ≥ 66th percentile) was used as dependent variables in logistic regression analyses. Variables with an association (*p* < 0.2) were examined as potential predictors of being in the highest 66th percentile (high iodine knowledge) and included in the initial multiple model. These were age, country of birth, study program, smoking habits, dry snuff habits, and thyroid disorder. Only variables with an association at *p* < 0.05 were kept in the model, using stepwise backwards selection conducted manually. Excluded variables were reintroduced one by one, to check for missed associations.

## 3. Results

Characteristics of the 403 participants and UIC by different characteristics are shown in Table 1. The median (p25–p75) age was 22 (21–25) years, and 62% were living in eastern Norway while 28% were living in western Norway. Most of the young women were students, of which 67% attended a health science study program and 24% attended a study program related to other sciences. About 9% of the women reported habitual use of iodine-containing supplements. The iodine content in the supplements ranged from 75–150 µg. Three women (1%) were currently planning a pregnancy, while 13% reported that they planned to become pregnant within two years.

The median (95% CI) UIC in the whole group was 75 (68, 84) µg/L and 31% had UIC < 50 µg/L, of which 20% were vegetarians and 80% non-vegetarians. Vegetarian dietary practices were reported by 36 (9%) of the women. Of these, 8 included milk and eggs in their diets (OL vegetarians), 15 included milk, eggs and fish (OLP vegetarians), 4 included eggs and fish (OP vegetarians), and 9 were vegans. UIC was significantly lower in vegetarians (all groups combined) than in non-vegetarians, with a median (p25–p75) UIC of 38 (25–55) µg/L in vegetarians and 80 (45–130) µg/L in non-vegetarians (*p* < 0.001).

**Figure 1:**
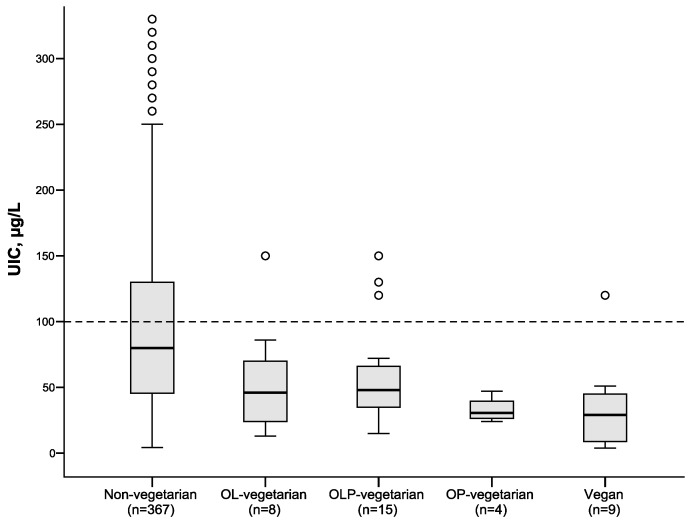
Urinary iodine concentration (UIC) among non-vegetarian women and among women with different vegetarian practices (ovo-lacto (OL), ovo-lacto-pesco (OLP), ovo-pesco (OP). Five observations with UIC > 350 µg/L are not shown in the non-vegetarian group. Box plot details: the horizontal lines indicate the median; the box indicates the interquartile range (IQR) (25th percentile to 75th percentile); the whiskers represent observations within 1.5 times the IQR and the circles are observations larger than 1.5 times the IQR. The stippled horizontal line marks the epidemiological criteria for assessing adequate iodine intake based on the median UIC by the World Health Organization [1].

Figure 1 illustrates the difference in UIC between non-vegetarians and different groups of vegetarians. As seen from the figure, vegans had the lowest median UIC (29 µg/L). Median UIC values in the other groups were as follows: ovo-pesco (OP) vegetarians—31 µg/L; ovo-lacto-pesco (OLP) vegetarians—48 µg/L; ovo-lacto (OL) vegetarians—46 µg/L; and non-vegetarians—80 µg/L.

UIC, habitual total iodine intake, and estimated iodine intake from UIC are shown in Table 2. Median (p25–p75) UIC was 75 (42–130) µg/L. The median (p25–p75) habitual iodine intake from food was 92 (68–122) µg/day and the median (p25–p75) total iodine intake from both food and supplements was 100 (69–136) µg/day. In supplement users, the median (p25–p75) iodine intake from supplements alone was 129 (43–150) µg/day and the total iodine intake was 200 (138, 256) µg/day. The median (p25–p75) estimated iodine intake from UIC was 117 (62–191) µg/day. 

**Figure 2:**
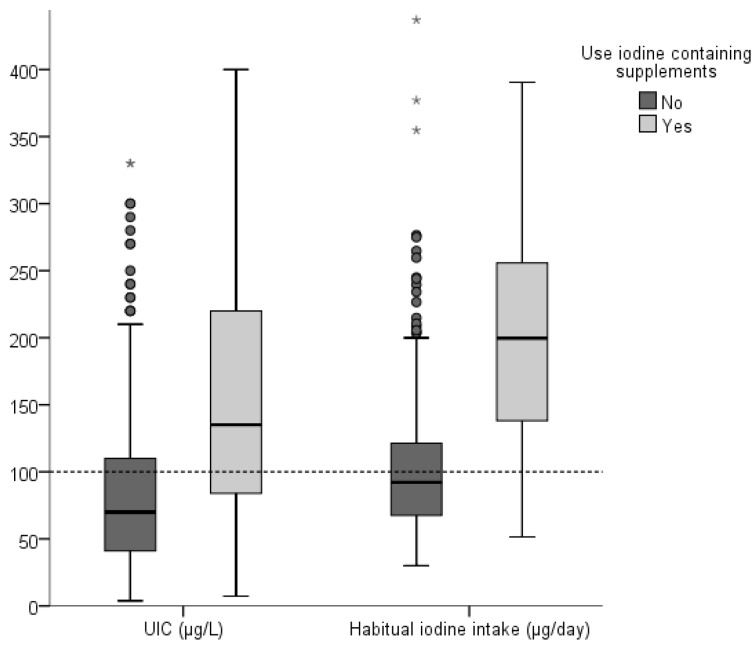
Urinary iodine concentration (UIC, µg/L) and habitual iodine intake (µg/day) in iodine supplement and non-supplement users. For Box plot details see Figure 1. The points are outliers and the stars, extreme outliers. The difference in UIC and habitual iodine intake between supplement and non-supplement users was significant (*p* < 0.001 for both) as tested by Mann Whitney U test. The stippled horizontal line marks the World Health Organization epidemiological criteria for adequate iodine intake based on the median UIC in children and non-pregnant adults [1] as well as the average requirement (AR) for adult women by the Nordic Nutrition Recommendations (NNR). The recommended daily intake in this group is 150 µg/day [26].

Both UIC and habitual iodine intake were significantly higher in iodine supplement users than in non-supplement users (Figure 2). Median (p25–p75) UIC was 135 (82–230) µg/L in iodine supplement users and 70 (41–115) µg/L in non-users. The median habitual iodine intake in was 200 (138–56) µg/day iodine supplement users and 92 (68–121) µg/L in non-users.

Spearman correlation between total iodine intake and UIC was 0.31 (95%CI: 0.22, 0.40) in all participants and 0.64 (95%CI: 0.40, 0.80) in iodine supplement users (*n* = 38). Quantile regression showed that vegetarian practice was associated with a reduction in median UIC of ~40 µg/L, while iodine supplement use increased the median UIC by ~70 µg/L. Other significant predictors for increased UIC were use of thyroid medication and iodine contributed by food (Table 3).

Answers to the questions about iodine knowledge in the young women are shown in Table 4. About 61% reported knowing what iodine is, 60% reported this within the health science fields, 65% within other sciences. The health science group did not have higher prevalence of correct answers regarding iodine sources, iodine functions, or iodine status in Norway. About half of the women correctly identified ‘milk and milk products’ (48%) and ‘fish and seafood’ (53%) as the most important dietary iodine sources in both science field groups. Dietary supplement use as a source of iodine was only correctly answered by 14%. Across both groups, 36% of the women were confident that they achieved the recommended daily intake of iodine through their diet, while 8% did not think they achieved the recommended daily intake, and 55% did not know.

The iodine knowledge scores according to study program is presented in Table 5. The median (p25–p75) score was 13 (10–17) points and 14 (11–17) points for health sciences and other sciences, respectively, on a scale from 0–24. There were 42% and 39% in the category of low knowledge score (7–12 points) among women in health sciences and other sciences, respectively. Forty percent and 48% had medium knowledge score (13–18 points) and 18% and 14% had high knowledge score (19–24 points) in the health sciences and other sciences, respectively.

Attributes associated with being in the highest tertile of the iodine knowledge score are presented in Table 6. As age increased, the probability of being in the upper tertile of the knowledge score increased, with adjusted odds ratio (95% CI) of 1.2 (1.1, 1.3). The women who reported to have a thyroid disorder had significantly higher probability for being in the upper tertile of the knowledge score than those who did not report any thyroid disorder, with an adjusted odds ratio (95% CI) of 3.8 (1.4, 10.7). Further, the women who were born outside Norway, had lower probability of being in the upper tertile of the knowledge score with an adjusted odds ratio (95% CI) of 0.4 (0.1, 1.0).

UIC was significantly higher in women in the two lowest tertiles (below the 66th percentile) of the knowledge score than in those in the upper 66th percentile (*p* = 0.005), with median (p25–p75) UIC of 83 (46–130) µg/L and 59 (32–110) µg/L, respectively. The habitual iodine intake was similar in the two knowledge score groups; the median (p25–p75) iodine intake was 95 (68–135) µg/day in the women below the 66th percentile and 104 (72–137) µg/day in those in the upper 66th percentile.

## 4. Discussion

Evaluation of median UIC and calculated iodine intake indicate that the young non-pregnant Norwegian women have insufficient iodine intake. This finding is in accordance with research published during the last 5 years which consistently documents mild to moderate iodine deficiency in different population groups in Norway [8,12,14,15,17,29,30]. The median cut-off for UIC at 100 µg/L to define adequate iodine intake in adult women is controversial, and a cut-off at 60–70 µg/L has been suggested [31,32]. Research is currently ongoing to better define the optimal median UIC range for non-pregnant women of reproductive age. However, there has been no change to the WHO guideline that no more than 20% of samples should be <50 μg/L [1]. Even with a lower median UIC cut-off, our results indicate insufficient iodine intake in the young women as 31% had UIC<50 µg/L. Of the 31% with UIC < 50 µg/L, 80% were non-vegetarians, showing that inadequate iodine status was not only a challenge among vegetarians. To the best of our knowledge, this is one of the first studies to evaluate iodine status and iodine knowledge in young non-pregnant nulliparous women. In a previous study in Norway assessing iodine status in population groups based on age and dietary practice, we found a median UIC of 71 µg/L in 51 non-pregnant women of childbearing age (18–45 years old) [15]. A study in UK schoolgirls reported a median UIC of 80 µg/L [10].

Inadequate iodine status in women of child-bearing age is of special concern since iodine deficiency may proceed into pregnancy [10]. An increasing number of observational studies have shown that mild to moderate iodine deficiency in pregnancy is associated with adverse neurocognitive outcomes in children [6,7,8,9]. Outcomes include language development, reading abilities, fine motor development and behavior problems. This if of high public health relevance as the consequences of suboptimal iodine nutrition incur high societal cost, while prevention of iodine deficiency has a low cost [1].

Calculated iodine intake in the young women was lower than the recommended daily intake. As milk is a main iodine source in countries without mandatory salt iodization, individuals who exclude milk and dairy products will be at high risk of insufficient iodine intake [17] unless they use an iodine containing supplement. According to the most recent national Norwegian dietary survey, young women (18–29 years old) had the lowest intake of milk and yoghurt compared to adult men and women [33]. Consumption of plant-based milk alternatives such as rice-, almond-and oat-milk is increasing, and none of these milk alternatives available in Norway contain iodine. Worldwide, as well as in Norway, there is an increasing number of individuals deliberately choosing a vegetarian diet or vegan diet [34]. In this study, vegetarian dietary practice was reported by 9% of the women and was a strong predictor for inadequate iodine status. Vegans (persons who exclude all animal derived food items) and ovo-pesco vegetarians (who exclude milk but include eggs and fish) had the lowest median UIC, followed by ovo-lacto vegetarians and ovo-lacto-pesco vegetarians. Many vegetarians are aware of the need to use iodine supplements, however, inadequate iodine status has been reported vegetarians in several studies and particularly in vegans [15,35,36,37]. With milk and white fish being the only available substantial iodine sources in the diet, it is obvious that individuals omitting these food items need to obtain iodine from supplements or from salt fortified with more iodine than what is currently available in Norway (currently 5 ppm and for household salt only).

We found that 9% of the young women reported using iodine-containing supplements. These women had substantially higher UIC and iodine intake than those who did not use iodine containing supplements, and reached the recommended daily iodine intake, contrary to what was found in a study of pregnant and lactating women, where iodine supplements did not contribute sufficient amounts of iodine for them to reach the recommended daily iodine intake [12,14]. Iodine-containing supplements should be initiated prior to pregnancy, as maternal thyroid hormones are important for the fetus during early gestation. Thus, correcting iodine deficiency even in early pregnancy may be too late to counteract the previous habitual low iodine intake [8,9].

Women with hypothyroidism are prescribed iodine-containing synthetic thyroid hormone medication (levothyroxine), which explains the positive association between use of thyroid disease medication and UIC. This finding is similar to what we found for pregnant women [12].

We found no difference in iodine status between students in the western and eastern region of Norway, as opposite to a recently published study [13], where regional differences in iodine status were observed for pregnant women. Traditionally, fish consumption has been higher in western than in eastern locations in Norway, however, today this is evident primarily in elderly individuals. Furthermore, the participants in our study were mainly students coming from all over the country to both study sites.

The level of iodine knowledge among the young Norwegian women was low and there was no difference between health science students and student on other science programs. Low iodine knowledge has also been reported among women in Australia [21], the UK, and Ireland [38] among adults in South Africa [39] and among students in the Philippines [40]. Increasing age, thyroid disorder and being born in Norway was associated with increased knowledge scores in our study. Contrary to this, younger age was positively associated with iodine knowledge among women of childbearing age in the UK and Ireland [38]. A positive association between thyroid disorder and iodine knowledge in our study might be explained by an increased awareness of iodine caused by the thyroid disease. Women born in another country than Norway had slightly lower iodine knowledge than women born in Norway; this may be explained by language challenges. Contrary to what could be expected, in our study, UIC was lower in women with high iodine knowledge than in those with low knowledge score. In a study of iodine status and awareness of iodine in the Philippines, no association was found between iodine status and knowledge [40]. However the study on women of childbearing age in the UK and Ireland found that iodine knowledge was associated with higher dietary iodine intake [38]. Approximately 40% of the young women in our study were aware that iodine deficiency might result in mental impairment. In comparison, this was reported by 43% of Australian women [41], 17% of Philippine students [40] and only 4% of the adult population in South Africa [39]. In South African adults [39] and Philippines students [40], only 15% and 37%, respectively, correctly identified iodized salt as the primary dietary source of iodine. In our study among young Norwegian women, in Australia [41], in UK and Ireland [38], 48%, 16%, and 9%, respectively, correctly identified milk and dairy products as important dietary iodine sources. Knowledge of iodine is of particular concern given the importance of good iodine status during pregnancy and lactation to prevent iodine deficiency disorders in the offspring.

Approximately 40% of the young women had low iodine knowledge scores, compared to 75% of pregnant women and 55% of lactating women, in a previous study in Norway [22]. The seemingly increased iodine knowledge in young women could be due to increased focus on iodine by the Norwegian Health authorities [16] and increased media attention on iodine during the last year. The authorities have recently updated the dietary recommendations on iodine for women of childbearing age. Women with a daily intake of less than three glasses of milk or yoghurt who consume little or no fish should take iodine-containing supplements [42]. Furthermore, a risk–benefit evaluation of increased salt iodization in household salt and salt used in industrially baked bread has been initiated [43]. In the meantime, it is important to increase awareness of iodine sources and how to obtain adequate iodine intake among health care workers, young women and the general population. 

The strengths of this study include a relatively large sample size with participants from two different geographical regions of Norway, in addition to data both on iodine intake and urinary iodine. The major limitation is the sampling procedure, which did not ensure that the participants are representative of all young women in Norway. Spot urine samples for assessment of UIC reflect recent, short time iodine intake. There is large inter- and intra-individual variation in UIC caused by differences in iodine intake as well as by large variation in fluid intake, but spot urine UIC is the recommended method for assessment of iodine status in groups [1,2]. Calculated habitual iodine intake from food and supplements may a better marker of iodine intake at the individual level, particularly in populations with few iodine sources. However, FFQs are relatively crude methods that are better suited for ranking individuals according to high and low intake than for precise estimation. The correlation of ~0.3 between the calculated habitual iodine intake and UIC in the whole group and of ~0.6 in iodine supplement users can be considered low to moderate agreement and shows that the frequency questions about foods and supplements provide a relatively valid estimate of iodine intake. Finally, urinary creatinine concentration was not assessed and we could not correct the UIC values for hydration status.

## 5. Conclusions

In conclusion, the present study shows that young Norwegian women have insufficient iodine intake and mild to moderate iodine deficiency. Furthermore, iodine status was substantially lower across all types of vegetarianism compared to non-vegetarians. The questions regarding iodine knowledge demonstrated a low to medium level of awareness about the dietary iodine sources and about the importance of iodine. Maternal iodine status is of particular importance for fetal development, and thus the implications of young women having low urinary iodine concentration and low iodine intake is of special concern. Public health strategies are needed to improve and secure adequate iodine intake in this vulnerable group.

## Figures and Tables

**Table 1:** Sample characteristics and median urinary iodine concentration (UIC) among young Norwegian women (*n* = 403).

Characteristics		UIC (µg/L) within Subgroups	
		Median (p25–p75)	*p*-value
Age, years	22.0 (21.0–24.0)		
BMI, kg/m^2^	22.6 (20.8–24.8)		
<18.5	15 (3.7)	86 (39–140)	0.409
18.5–24.9	291 (72.2)	72 (41–120)	
≥25.0–29.9	71 (17.6)	88 (55–130)	
≥30	21 (5.2)	72 (44–150)	
Geographic area			
Eastern Norway	250 (62.0)	75 (42–130)	0.735
Western Norway	114 (28.3)	75 (42–110)	
Country of birth			
Norway	373 (92.6)	74 (41–130)	0.405
Other	30 (7.4)	85 (54–123)	
Relationship status			
Married/in a relationship	192 (47.6)	74 (41–118)	0.450
Single	205 (50.9)	75 (45–130)	
Other	2 (0.5)	nd	
Health sciences ^a^	271 (67.2)	78 (42–130)	0.106
Other sciences ^b^	132 (23.8)	63 (42–110)	
Smoker			
Yes	24 (6.0)	69 (33–97)	0.151
No	379 (94.0)	75 (43–130)	
Dry snuff			
Yes	116 (28.8)	83 (39–130)	0.729
No	286 (71.0)	74 (43–120)	
Thyroid disease medication (self-reported) ^c^			
Yes	10 (4.2)	91 (53–135)	0.250
No	393 (95.8)	74 (42–130)	
Use of iodine-containing supplements ^d^			
Yes	38 (9.4)	135 (82–230)	<0.001
No	365 (90.6)	70 (41–115)	
Vegetarian ^e^			
Yes	36 (8.9)	38 (27–55)	<0.001
No	367 (90.8)	80 (45–130)	

Values given as median (p25–p75) or *n* (%). nd: not determined. Differences in UIC between groups were tested by the Mann Whitney U test or Kruskall Walllis, categories where UIC was nd were not included in the statistical tests. ^a^ Includes nutrition, public health nutrition, nursing, public health (nursing), midwifery, social nurse, physiotherapy, occupational therapy, mental healthcare ^b^ Includes biotechnology, chemistry, environmental sciences, engineering, physics, teaching, psychology, and other. ^c^ Thyroid disease includes goiter (*n* = 1), hyperthyroidism (*n* = 3) and hyperthyroidism (*n* = 13). ^d^ Habitual use. ^e^ Includes ovo-lacto vegetarians (*n* = 8), ovo-lacto-pesco vegetarians (*n* = 15), ovo-pesco vegetarians (*n* = 4), and vegans (*n* = 9). One missing from age; 4 missing from BMI; 2 missing from planning pregnancy currently; 3 missing from planning pregnancy within 2 years; 4 missing from relationship status; 1 missing from study program; 1 missing from dry snuff.

**Table 2:** Urinary iodine concentration and habitual iodine intake from food and supplements in young Norwegian women (*n* = 403).

	Mean ± SD	Median	p25	p75
Urinary iodine concentration, µg/L	94 ± 76	75	42	130
Habitual iodine intake				
Iodine from food, µg/day	104 ± 58	92	68	122
Iodine from supplements (users only) ^a^, µg/day	106 ± 60	129	43	150
Total iodine intake, µg/day	114 ± 68	100	69	135
Estimated iodine intake from UIC ^b^, µg/day	149 ± 125	117	62	191

^a^ Supplement users (*n* = 38) ^b^ Estimated iodine intake from UIC were calculated using the following equation: UIC × 0.0235 × weight (kg) [28].

**Table 3:** Predictors for change in median urinary iodine concentration (UIC, µg/L)^a^ in young Norwegian women (*n* = 403).

Predictor Variables	Unadjusted Coeff. (95% CI)	*p*	Adjusted Coeff. (95% CI)	*p*
Constant			57 (44, 70)	<0.001
Vegetarian ^b^	−42 (−65, −18)	<0.001	−37 (−53, −21)	<0.001
Iodine supplement user ^c^	70 (47, 93)	<0.001	68 (45, 91)	<0.001
Use of thyroid medication ^d^	36 (−13, 85)	0.146	23 (6, 40)	0.008
Iodine intake from food, 100 µg	23 (10, 34)	<0.001	18 (8, 2)	0.001

^a^ Median regression analysis (quantile regression) with non-transformed UIC as the dependent variable, ^b^ Includes all vegetarian groups (0 = no, 1 = yes), ^c^ Habitual iodine supplement use vs non-use, (0 = no, 1 = yes). ^d^ Reported use of medication for thyroid disease, (0 = no, 1 = yes). The adjusted model included the four predictors.

**Table 4:** Iodine knowledge in young Norwegian women attending health related study programs and other study programs (*n* = 403) ^a.^

Iodine Knowledge	Health Sciences ^b^ (*n* = 271)	Other Sciences ^c^ (*n* = 132)	*p*	All (*n* = 403)
Do you know what iodine is?				
No	111 (41.1)	46 (34.8)	0.271	157 (39.1)
Yes	159 (58.9)	86 (65.2)		245 (60.9)
Most important dietary iodine sources ^#^				
Meat	56 (20.9)	25 (18.9)	0.754	81 (20.1)
Milk and milk products *	128 (47.8)	67 (50.8)	0.647	195 (48.4)
Fruit and vegetables	21 (7.8)	22 (16.7)	0.012	43 (10.7)
Fish and seafood *	146 (54.5)	68 (51.5)	0.651	214 (53.1)
Bread	34 (12.7)	15 (11.4)	0.828	49 (12.2)
Vegetable oil	5 (1.9)	2 (1.5)	nd	7 (1.7)
Iodized salt	37 (51.1)	62 (47.0)	0.500	199 (49.4)
Dietary supplements *	37 (13.9)	18 (13.6)	1.000	55 (13.6)
Don’t know	64 (23.9)	26 (19.7)	0.415	90 (22.3)
Iodine is important for: ^#^				
Child growth and development *	103 (38.6)	66 (50.0)	0.039	169 (41.9)
Preventing blindness	11 (4.1)	2 (1.5)	nd	13 (3.2)
Normal fetal development *	100 (37.5)	40 (30.3)	0.195	140 (34.7)
Strength in teeth and skeleton	41 (15.4)	24 (18.2)	0.565	65 (16.1)
Maintaining a normal metabolism *	117 (43.8)	54 (40.9)	0.656	171 (42.4)
Preventing spina bifida	11 (4.1)	3 (2.3)	nd	14 (3.5)
Don’t know	87 (32.6)	44 (33.6)	0.931	131 (32.5)
Iodine status in Norway ^#^				
Too low intake is a current problem *	72 (26.6)	42 (31.8)	0.423	114 (28.3)
Too high intake is a current problem	12 (4.4)	3 (2.3)	nd	15 (3.7)
Too low intake was only a problem earlier	29 (10.7)	21 (15.9)	0.224	50 (12.4)
Don’t know	149 (55.0)	66 (50.0)	0.252	215 (53.3)
I think I get enough iodine through the diet				
Agree	101 (37.7)	43 (32.6)	0.373	144 (35.7)
Disagree	22 (8.2)	12 (9.1)	0.915	34 (8.4)
Don’t know	145 (54.1)	77 (58.3)	0.488	222 (55.1)

* Correct answer, ^#^ Multiple answers possible. 1 missing from “Do you know what iodine is”; 3 missing from “iodine sources”; 4 missing from “functions of iodine”; 8 missing from “iodine status in Norway”; 3 missing from “perception of own iodine intake”; ^a^ Values are presented as n (%) for all, and *n* (%) within study direction for health sciences and other sciences. nd = not determined due to low cell count. ^b^ Health sciences include: nutrition, public health nutrition, nursing, public health (nursing), midwifery, social nurse, physiotherapy, occupational therapy, mental healthcare.^c^ Other sciences include: biotechnology, chemistry, environmental sciences, engineering, physics, teaching, psychology, and other (*n* = 11)/working (*n* = 8).

**Table 5:** Iodine knowledge scores in young Norwegian women attending health-related study programs and other study programs (*n* = 392) ^a.^

Iodine Knowledge Score	Health Sciences (*n* = 260)	Other Sciences (*n* = 132)	All (*n* = 392)
Total score ^b^	13 (10–17)	14 (11–17)	14 (10–17)
Poor	1 (0.4)	0	1 (0.3)
Low	109 (41.9)	51 (38.6)	160 (40.8)
Medium	104 (40.0)	63 (47.7)	167 (42.6)
High	46 (17.7)	18 (13.6)	64 (16.3)

^a^ Values are presented as median (p25–p75) *n* (%) for all, and n (%) within study direction for health sciences and other sciences. ^b^ Scores range is from 0 to 24. Poor = 0–6 points; low = 7–12 points; medium = 13–18 points; high = 19–24 points. Differences in total score were tested with the Mann Whitney U test (*p* = 0.498).

**Table 6:** Attributes associated with being in the highest tertile of knowledge score among young Norwegian women (*n* = 392) ^a^.

	Unadjusted Coefficients			Adjusted Coefficients		
	OR	95% CI	*p*	OR	95% CI	*p*
Age	1.2	1.1, 1.3	<0.001	1.2	1.1, 1.3	<0.001
Thyroid disorder ^b^	3.7	1.3, 10.1	0.012	3.8	1.4, 10.7	0.011
Birth country ^c^	0.4	0.1, 1.0	0.048	0.4	0.1, 1.0	0.049

^a^ One missing from age; *n* = 391 in the multiple model and for age. ^b^ Self-reported thyroid disorder, including hypo- and hyperthyroidism and goiter. Categories for thyroid disorder: 0 = No, 1 = yes. ^c^ Categories for birth country: 0 = Norway, 1 = Other country.
